# Data integration into national health information systems: the Ethiopia national trachoma control programme

**Published:** 2022-03-01

**Authors:** Amsayaw Tefera, Fikre Seife, Alexandre Pavluck, Biruck Kebede

**Affiliations:** 1 Federal Ministry of Health, Addis Ababa, Ethiopia.; 2 Sightsavers, Atlanta, GA, USA.; 3 RTI International, Addis Ababa, Ethiopia.

**The new electronic community health information system will avoid reporting delays and promote investment.**

**Figure:**
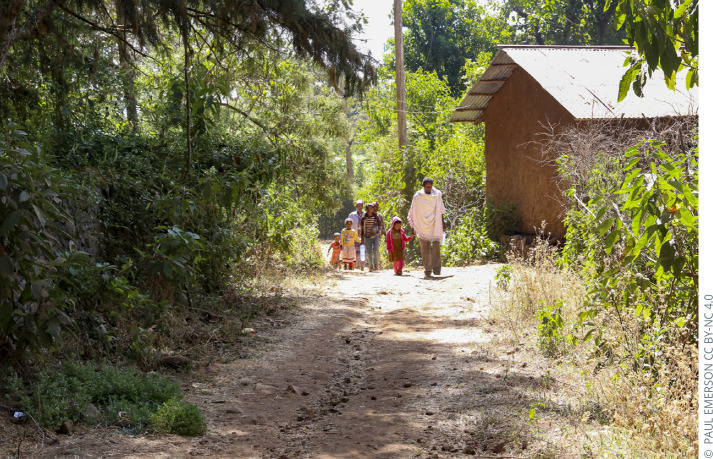
Some Ethiopians must walk long distances to reach medical treatment for trachoma. **ETHIOPIA**

Globally, significant progress has been made towards the elimination of trachoma as a public health problem. Since 2002, the scale-up of all components of the WHO-endorsed SAFE strategy (surgery, antibiotics, facial cleanliness, environmental improvements) has contributed to a 91% reduction in the number of people at risk from trachoma, from 1.5 billion to 136.2 million today.^1^

As the global trachoma programme matures, increased attention is now being placed on the sustainability of national programmes establishing case identification and surveillance, integrating services within the routine health system, maintaining the quality of services, and ensuring no one is left behind. To achieve these goals, the global trachoma programme is transitioning away from donor-led disease-specific models of programme delivery towards country-owned and integrated delivery of trachoma interventions through national eye health systems.

In Ethiopia, which accounts for 49% of the global burden of trachoma, neglected tropical diseases (NTDs), including trachoma, are neglected in part because the health care burden due to these diseases is under-represented in the data used by the national health system. This means that NTDs are not part of national health management systems so when those systems are being used to set national health priorities and investment, NTDs are either not available in those systems or only partially available. Recognising this, in 2017, the Ethiopian Federal Ministry of Health invested in the expansion of NTD indicators – including mass drug administration (MDA) and morbidity management for trachoma – in the newly designed national health management information system (HMIS).

Ethiopia's national trachoma programme is also moving away from paper-based, disease-focused vertical reporting for MDA to an electronic community health information system (eCHIS) which is linked to the national HMIS. This is being done in a step-by-step process where, for instance, trachoma data are reported through both the integrated (HMIS) data flow and the disease-focused vertical data flow collected and reported by NTD staff at the different levels. Data provided by both systems are compared to highlight areas where more training on NTD data recording and reporting through HMIS is needed. Once the integrated data flow is working sufficiently, measured by accuracy in reporting the true figure of treatments delivered, the vertical data flow will be discontinued, leaving the integrated data flow as the sole source for reporting programmatic data. This integrated system avoids manual, logical, and transcription errors as well as reporting delays and promotes further investment in strengthening national electronic data register and reporting systems, thereby ultimately benefiting all health programmes and creating a more resilient health system.

Aligned with what has already been started in Ethiopia in the past few years, the WHO NTD road map (2021–2030)^2^ emphasises the transition of NTD data into national health information systems to establish country-led, integrated programmes. The implementation of this guidance is critical to the sustainability of programmes and is being supported by three new transition toolkits^3^ developed by the International Coalition for Trachoma Control, which show a successful transition should lead to validation of elimination and confidence that all cases will be managed properly in a post-elimination setting.

Transition will undoubtedly require increased partnership, including clear guidance and support from donors and implementing partners, and learning from experiences in other countries. However, its benefits will ensure that today's investments in trachoma elimination are sustained into the future through a comprehensive and strengthened eye health system.

## References

[1] World Health Organization. WHO Alliance for the Global Elimination of Trachoma by 2020: progress report on elimination of trachoma, 2020. Weekly Epidemiological Record 2021;96(31): 353–364. bit.ly/WHO-WER21

[2] World Health Organization. Ending the neglect to attain the Sustainable Development Goals: a road map for neglected tropical diseases 2021-2030. bit.ly/WHOntdmap

[3] International Coalition for Trachoma Control (ICTC) toolkits: Transition planning for facial cleanliness and environmental improvement, mass drug administration, and trichiasis management services. Available from bit.ly/transition-toolkits

